# Supplementary material for: Dauer fate in a Caenorhabditis elegans Boolean network model
Source: PeerJ. 2023 Jan 23;11:e14713. doi: 10.7717/peerj.14713 (PMC9879150; doi:10.7717/peerj.14713)
Supplement: Table S1 [file peerj-11-14713-s001.docx]

**Supplementary Table 1** Regulatory interactions of the dauer Boolean network.

| **Upstream node** | **Downstream node** | **Interaction** | **PubMed ID** |
| --- | --- | --- | --- |
| *aap-1* | *age-1* | Activating | 12393910 |
| *age-1* | *pdk-1* | Activating | 10364160 |
| *akt** | *daf-16* | Inhibitory | 11747825, 11381260, 11747821, 9716402 |
| *cmk-1* | *daf-28* | Activating | 26335407 |
| *cmk-1* | *daf-7* | Activating | 26335407 |
| *daf-1* | *daf-8-14** | Activating | 10887089 |
| *daf-11** | *tax-4* | Activating | 8893027 |
| *daf-12* | *daf-16* | Activating | 15383841, 12966085 |
| *daf-12* | *daf-16* | Activating | 15383841 |
| *daf-12* | *daf-9* | Activating | 15084461 |
| *daf-12* | dauer | Activating | 15383841 |
| *daf-16* | *ins-18* | Activating | 22683638 |
| *daf-16* | *ins-7* | Inhibitory | 12845331 |
| *daf-16* | *ins-7* | Inhibitory | 19489741 |
| *daf-16* | dauer | Activating | 15383841 |
| *daf-2* | *aap-1* | Activating | 12393910 |
| *daf-2* | *dhs-16* | Activating | 22505847 |
| *daf-2* | *hsf-1* | Inhibitory | 23116063 |
| *daf-28* | *daf-2* | Activating | 12654727, 27125673 |
| *daf-3* | *daf-5* | Activating | 14681186 |
| *daf-5* | *ins-1* | Activating | 21533078 |
| *daf-7* | *daf-1-4* | Activating | 10887089, 8413626, 8570636 |
| *daf-7* | *ins-7* | Activating | 19489741 |
| *daf-8-14** | *daf-3* | Inhibitory | 20081192, 14992718 |
| *dhs-16* | *daf-9* | Inhibitory | 22505847 |
| *hsf-1* | *daf-7* | Inhibitory | 23116063 |
| *ins-1* | *daf-2* | Inhibitory | 21343369, 11274053 |
| *ins-18* | *daf-2* | Inhibitory | 22683638 |
| *ins-7* | *daf-2* | Activating | 12845331 |
| *ncr** | *daf-9* | Activating | 15509773 |
| *pdk-1* | *akt** | Activating | 10364160 |
| *pher** | *srbc** | Activating | 19797623 |
| *srbc** | *daf-11** | Inhibitory | 19797623 |
| *tax-4* | *daf-7* | Activating | 16903785 |

*****The nodes names used in literature have been modified for convenience. Here, *daf-11* = cGMP, pher = Pheromone, *ncr* = *ncr-1/2,* *daf-8-14* = *daf-8* and *daf-14*, *akt* = *akt-1/2*, *srbc* = *srbc-64* and *srbc-66*.
