# Supplementary material for: Dauer fate in a Caenorhabditis elegans Boolean network model
Source: PeerJ. 2023 Jan 23;11:e14713. doi: 10.7717/peerj.14713 (PMC9879150; doi:10.7717/peerj.14713)
Supplement: Table S2 — Initial conditions for dauer were cmk-1 = 0, ncr = 0 and pher = 1. Initial conditions for normal development were cmk-1 = 1, ncr = 1 and pher = 0. The state of each node at time ‘t+1’ was calculated according to the states of upstream regulator nodes at time ‘t’. [file peerj-11-14713-s002.docx]

**Supplementary Table 2** The Boolean rules of the dauer network. Initial conditions for dauer were *cmk-1* = 0, *ncr* = 0 and pher = 1. Initial conditions for normal development were *cmk-1* = 1, *ncr* = 1 and pher = 0. The state of each node at time ‘t+1’ was calculated according to the states of upstream regulator nodes at time ‘t’.

**Downstream node *= Upstream regulators**

*aap-1 *= daf-2*

*age-1 *= aap-1*

*akt *= pdk-1*

*daf-11 *=* not *srbc*

*daf-12 *=* not *daf-9* or not *daf-7*

*daf-1-4 *= daf-7*

*daf-16 *=* not *akt* or *daf-12*

*daf-2 *= (ins-7* and *daf-28)* or *(*not *ins-1* or not *ins-18)*

*daf-28 *= cmk-1*

*daf-3 *=* not *daf-8-14*

*daf-5 *= daf-3*

*daf-7 *= (tax-4* and *cmk-1)* and not *hsf-1*

*daf-8-14 *= daf-1-4*

*daf-9 *=* not *dhs-16* or *ncr* or *daf-12*

*dauer *= daf-16* and *daf-12*

*dhs-16 *= daf-2*

*hsf-1 *=* not *daf-2*

*ins-1 *= daf-5*

*ins-18 *= daf-16*

*ins-7 *= daf-7* or not *daf-16*

*pdk-1 *= age-1*

*srbc *= pher*

*tax-4 *= daf-11*
