# Supplementary material for: Dauer fate in a Caenorhabditis elegans Boolean network model
Source: PeerJ. 2023 Jan 23;11:e14713. doi: 10.7717/peerj.14713 (PMC9879150; doi:10.7717/peerj.14713)
Supplement: Table S3 [file peerj-11-14713-s003.docx]

**Supplementary Table 3** Stable motifs in the dauer Boolean network.

| **Name** | **Stable motif** | **Perturbation** |
| --- | --- | --- |
| SM 4 | *{'ins-18': 1, 'pdk-1': 0, 'daf-5': 1, 'akt': 0, 'age-1': 0, 'daf-16': 1, 'aap-1': 0, 'hsf-1': 1, 'daf-3': 1, 'daf-8-14': 0, 'daf-2': 0, 'ins-1': 1, 'daf-7': 0, 'daf-1-4': 0}* | *ins-7*=0 |
| SM 5 | *{'ins-18': 1, 'daf-5': 1, 'daf-12': 1, 'daf-16': 1, 'hsf-1': 1, 'daf-3': 1, 'daf-8-14': 0, 'daf-2': 0, 'ins-1': 1, 'daf-7': 0, 'daf-1-4': 0}* | *ins-7*=0 |
| SM 6 | *{'pdk-1': 0, 'daf-5': 1, 'akt': 0, 'age-1': 0, 'daf-16': 1, 'aap-1': 0, 'hsf-1': 1, 'daf-3': 1, 'daf-8-14': 0, 'daf-2': 0, 'ins-1': 1, 'daf-7': 0, 'ins-7': 0, 'daf-1-4': 0}* | *ins-18*=1 |
| SM 7 | *{'daf-5': 1, 'daf-12': 1, 'daf-16': 1, 'hsf-1': 1, 'daf-3': 1, 'daf-8-14': 0, 'daf-2': 0, 'ins-1': 1, 'daf-7': 0, 'ins-7': 0, 'daf-1-4': 0}* | *ins-18*=1 |
| SM 8 | *{'daf-5': 1, 'daf-12': 1, 'daf-16': 1, 'hsf-1': 1, 'daf-3': 1, 'daf-8-14': 0, 'daf-2': 0, 'ins-1': 1, 'daf-7': 0, 'ins-7': 0, 'daf-1-4': 0}* | *daf-12*=1, *daf-16*=1, *akt*=0, *pdk-1*=0, *age-1*=0, *aap-1*=0 |
| SM 9 | *{'ins-18': 1, 'daf-12': 1, 'daf-16': 1, 'daf-2': 0, 'daf-7': 0, 'ins-7': 0, 'hsf-1': 1}* | *ins-1*=1, *daf-5*=1, *daf-3*=1, *daf-8-14*=0, *daf-1-4*=0 |
| SM 10 | *{'ins-18': 1, 'pdk-1': 0, 'akt': 0, 'age-1': 0, 'daf-16': 1, 'hsf-1': 1, 'daf-2': 0, 'daf-7': 0, 'ins-7': 0, 'aap-1': 0}* | *ins-1*=1, *daf-5*=1, *daf-3*=1, *daf-8-14*=0, *daf-1-4*=0 |
