# Supplementary figures and images for: Dauer fate in a Caenorhabditis elegans Boolean network model
Source: PeerJ. 2023 Jan 23;11:e14713. doi: 10.7717/peerj.14713 (PMC9879150; doi:10.7717/peerj.14713)

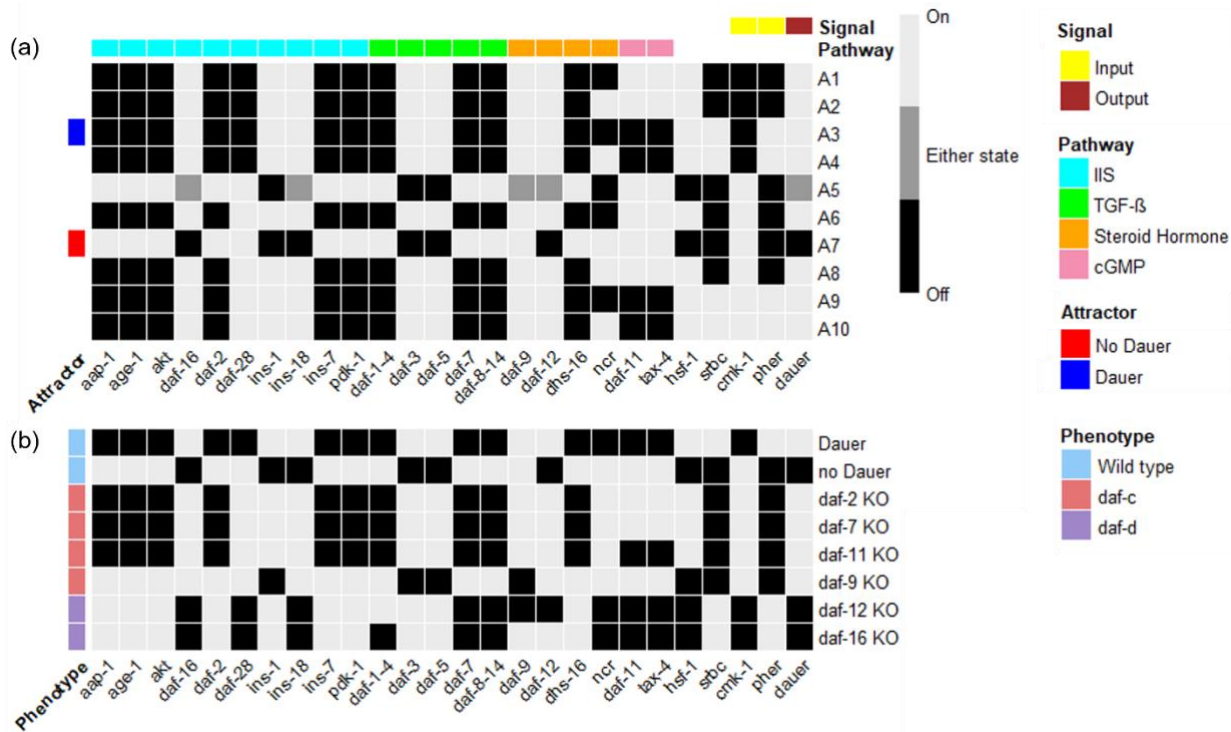

Supplement: Figure S1 — (A) A heatmap that shows the states of every node of the system in its steady state for 10 different attractors (A1 –A10) identified for this dauer network. Attractors A3 and A7 represent the two wild type phenotypes of this system i.e. “dauer” and “no dauer”. The nodes part of the four different pathways and those that are output/input signals are annotated. (B) A similar heatmap for different phenotypes exhibited by the system. [file peerj-11-14713-s004.pdf]
