# Supplementary material for: Dauer fate in a Caenorhabditis elegans Boolean network model
Source: PeerJ. 2023 Jan 23;11:e14713. doi: 10.7717/peerj.14713 (PMC9879150; doi:10.7717/peerj.14713)

Simulation Trajectories for wildtype models


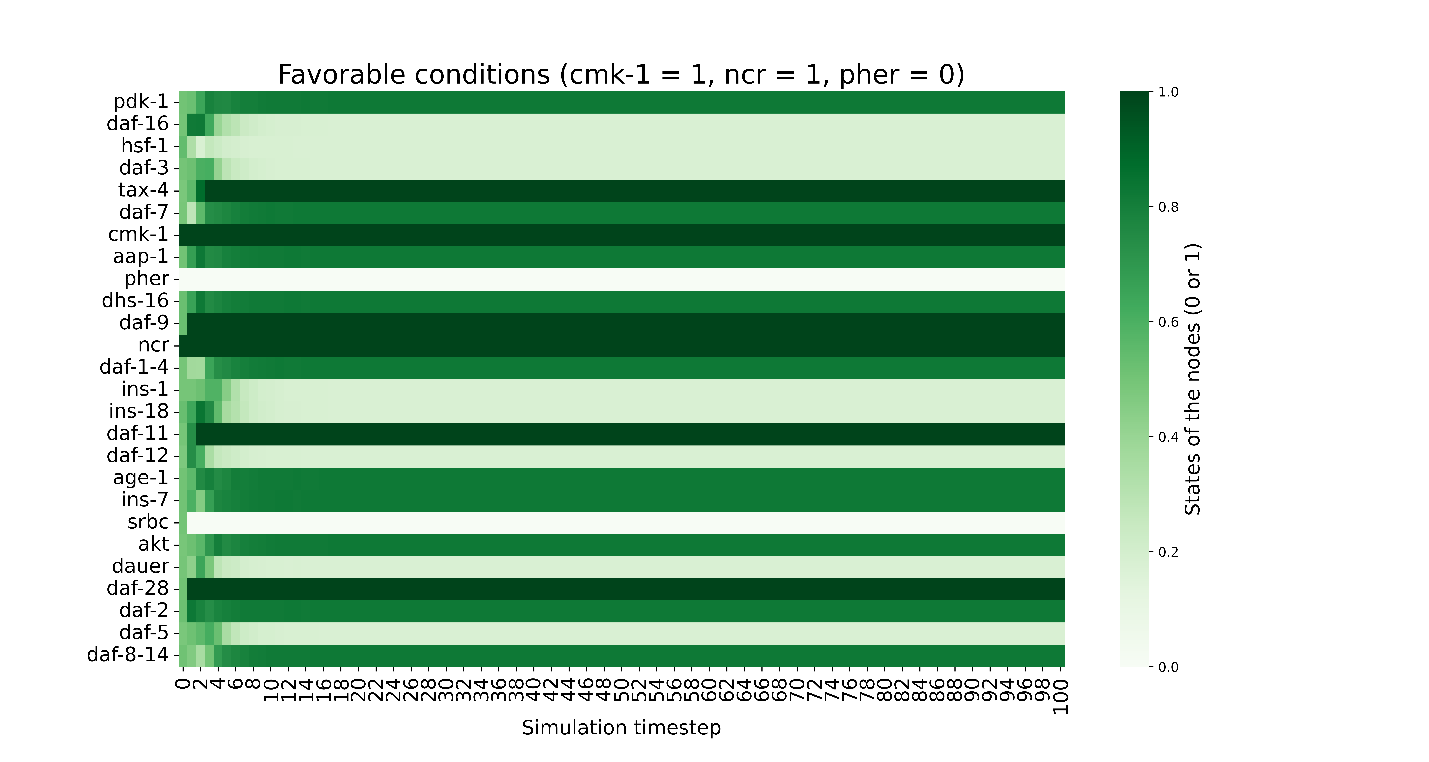


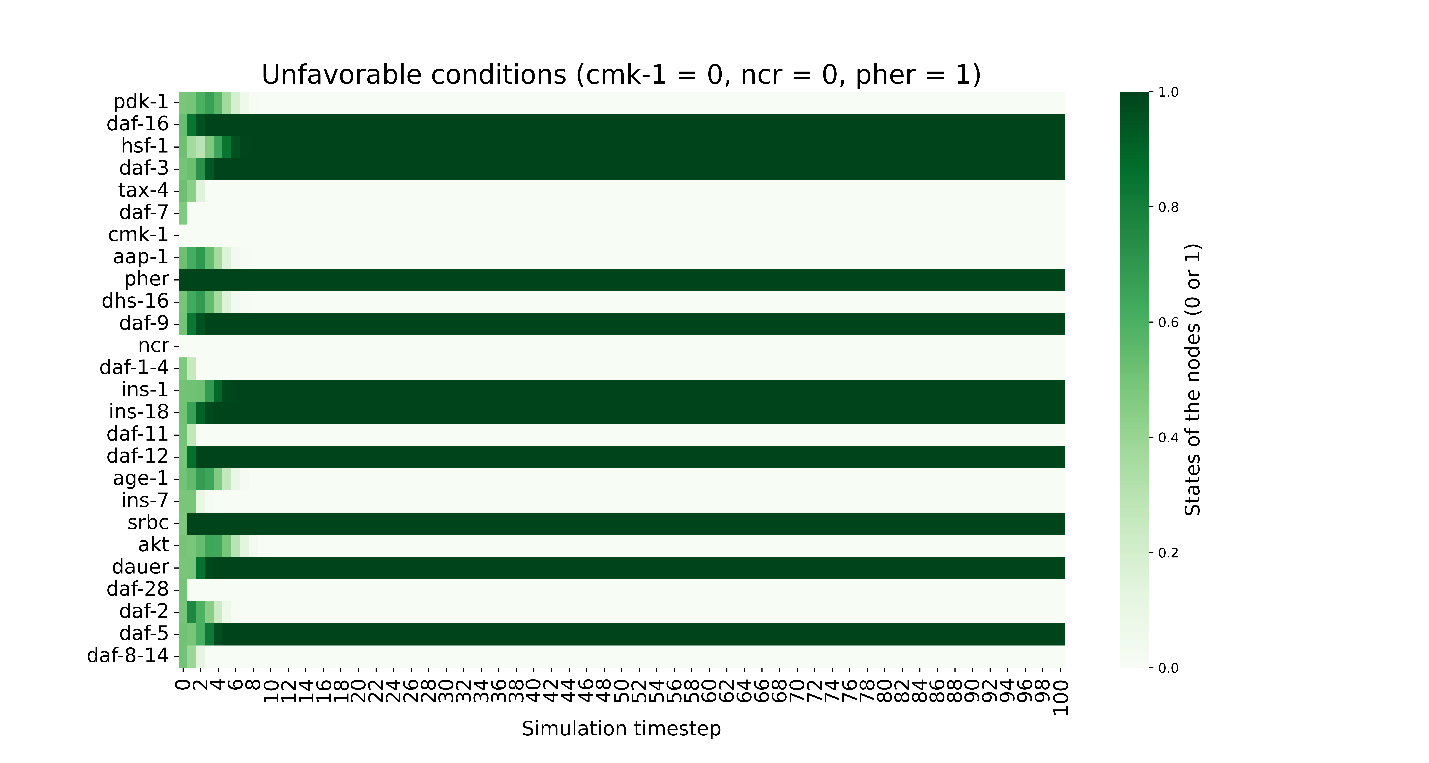


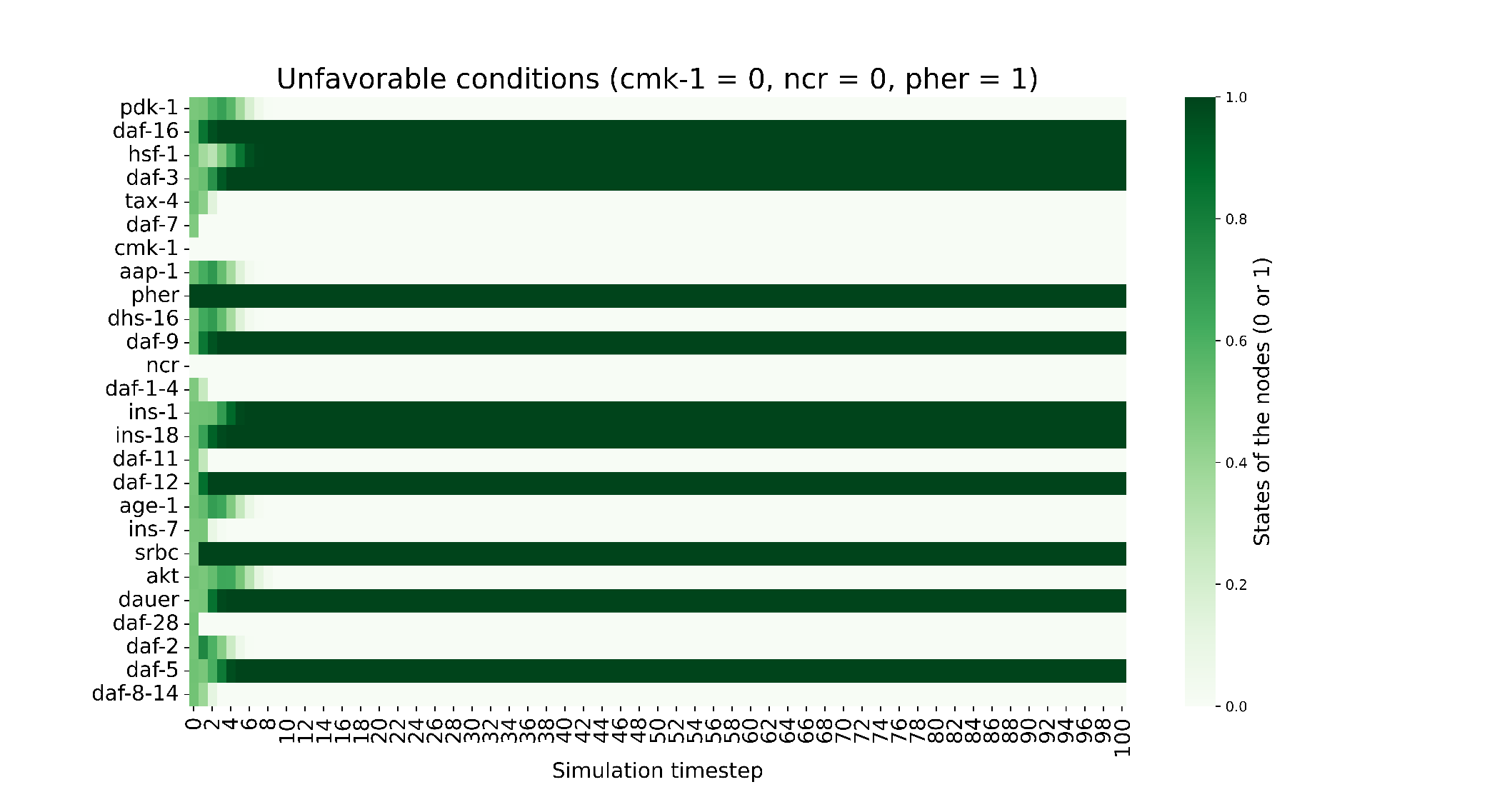


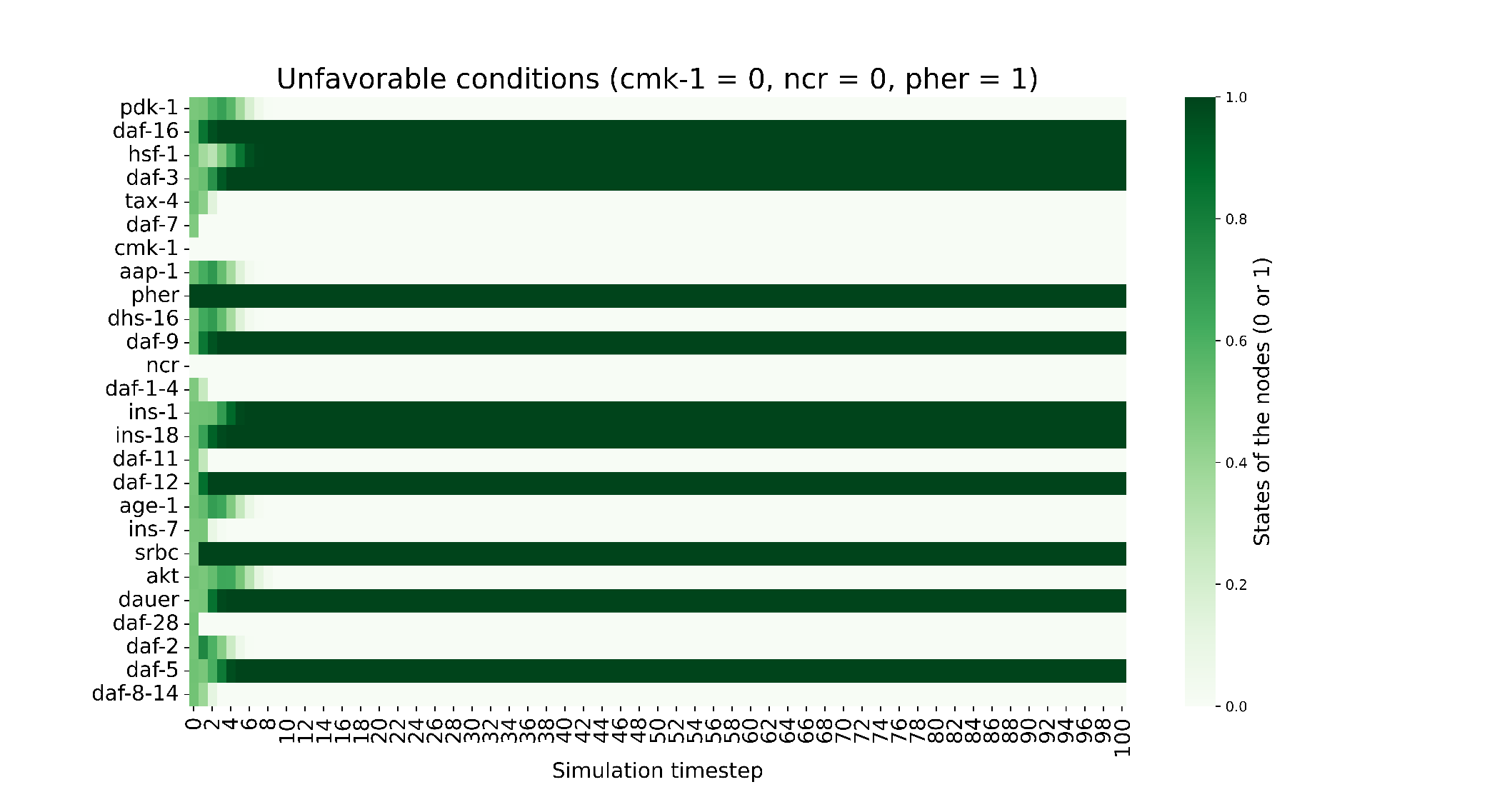

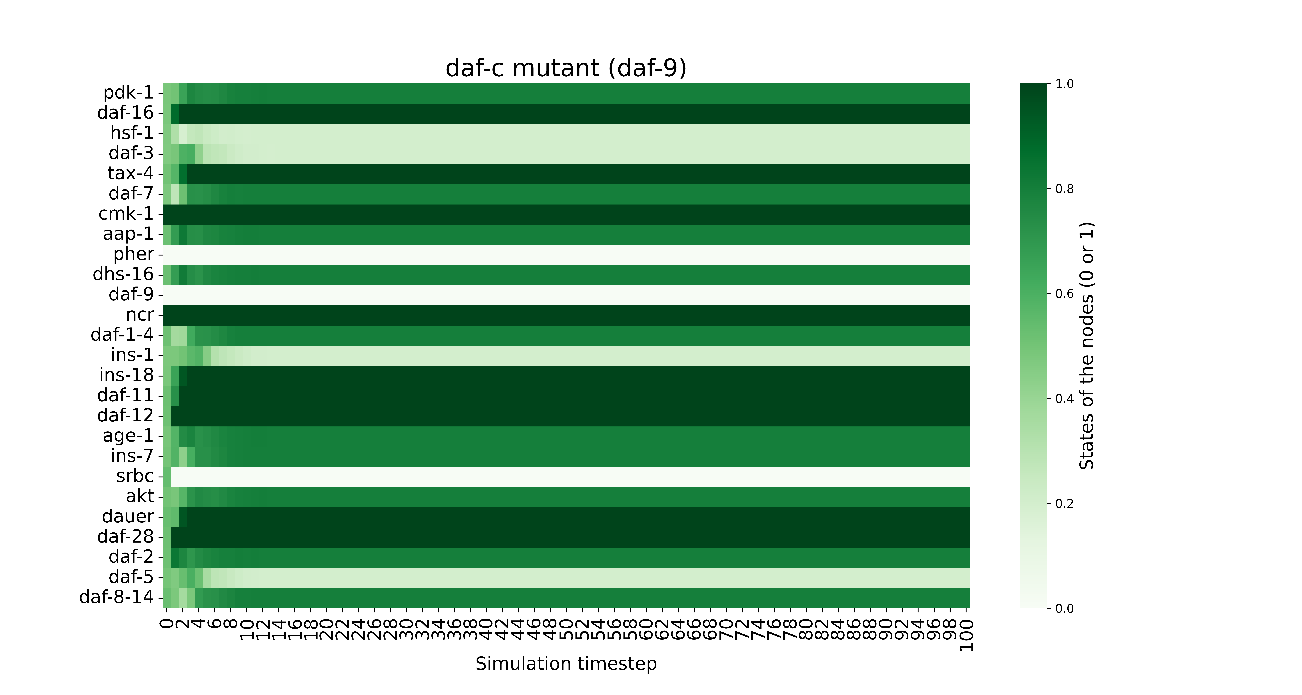

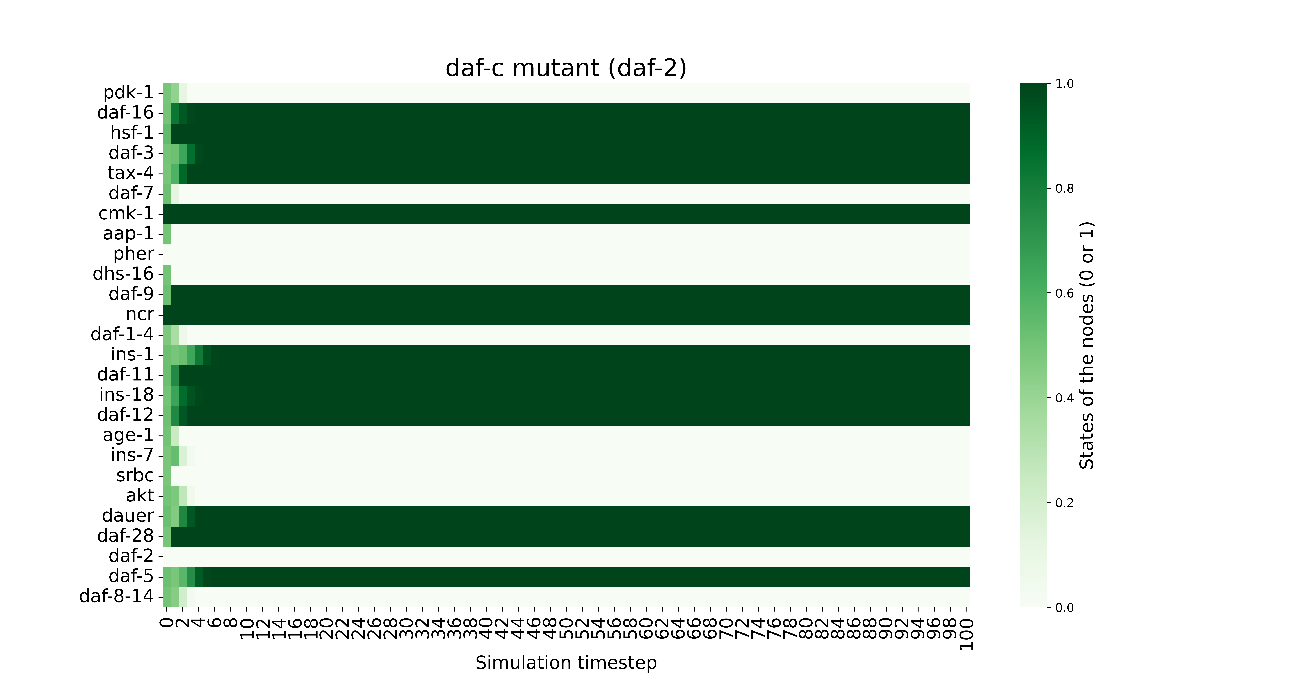

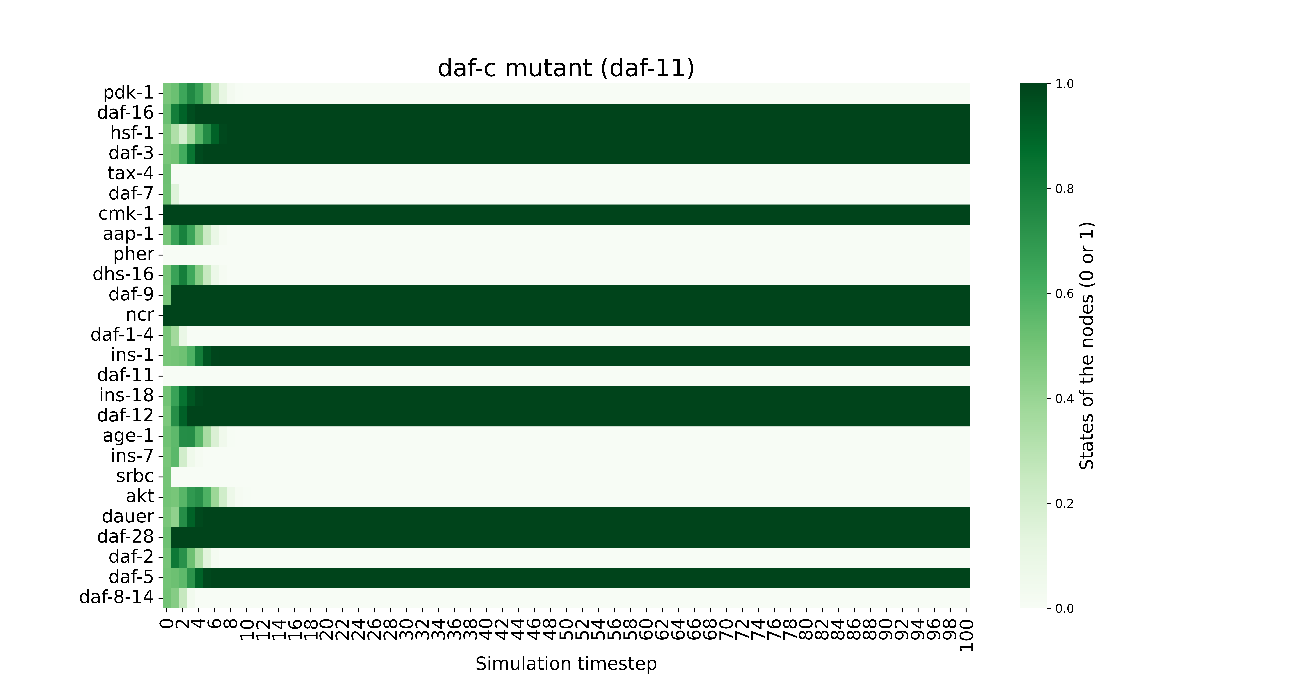
Simulation Trajectories for daf-c mutant models


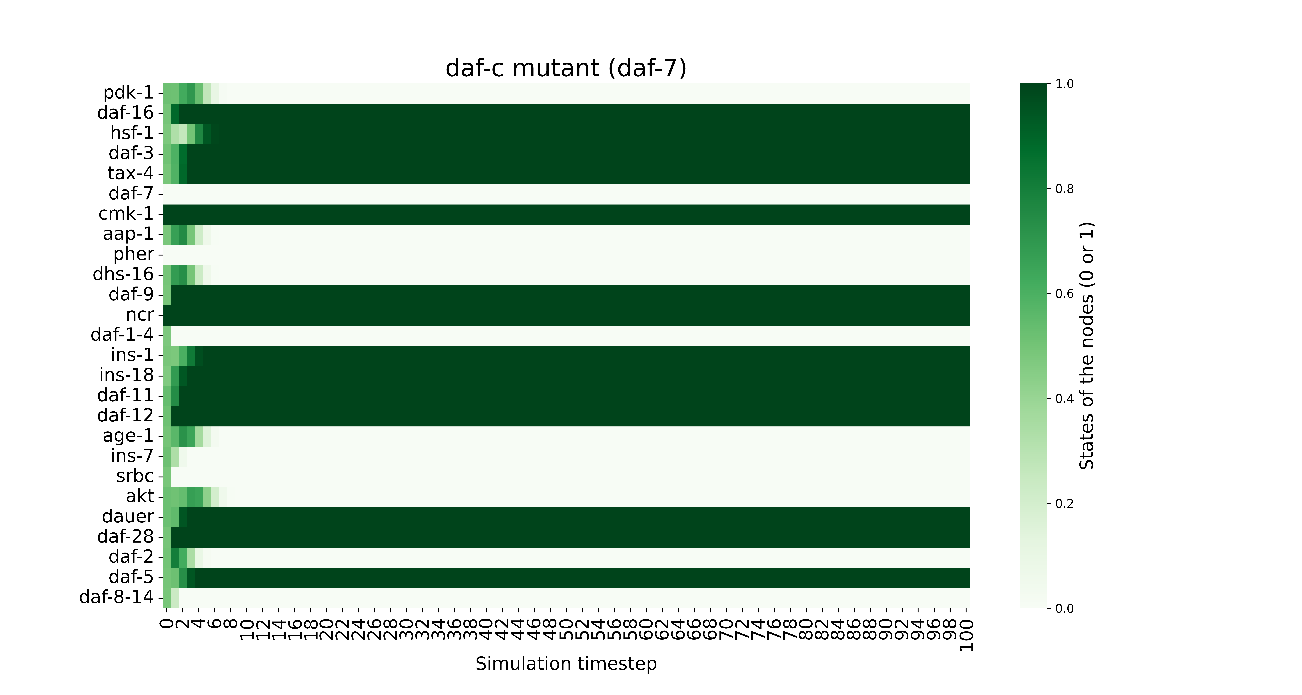


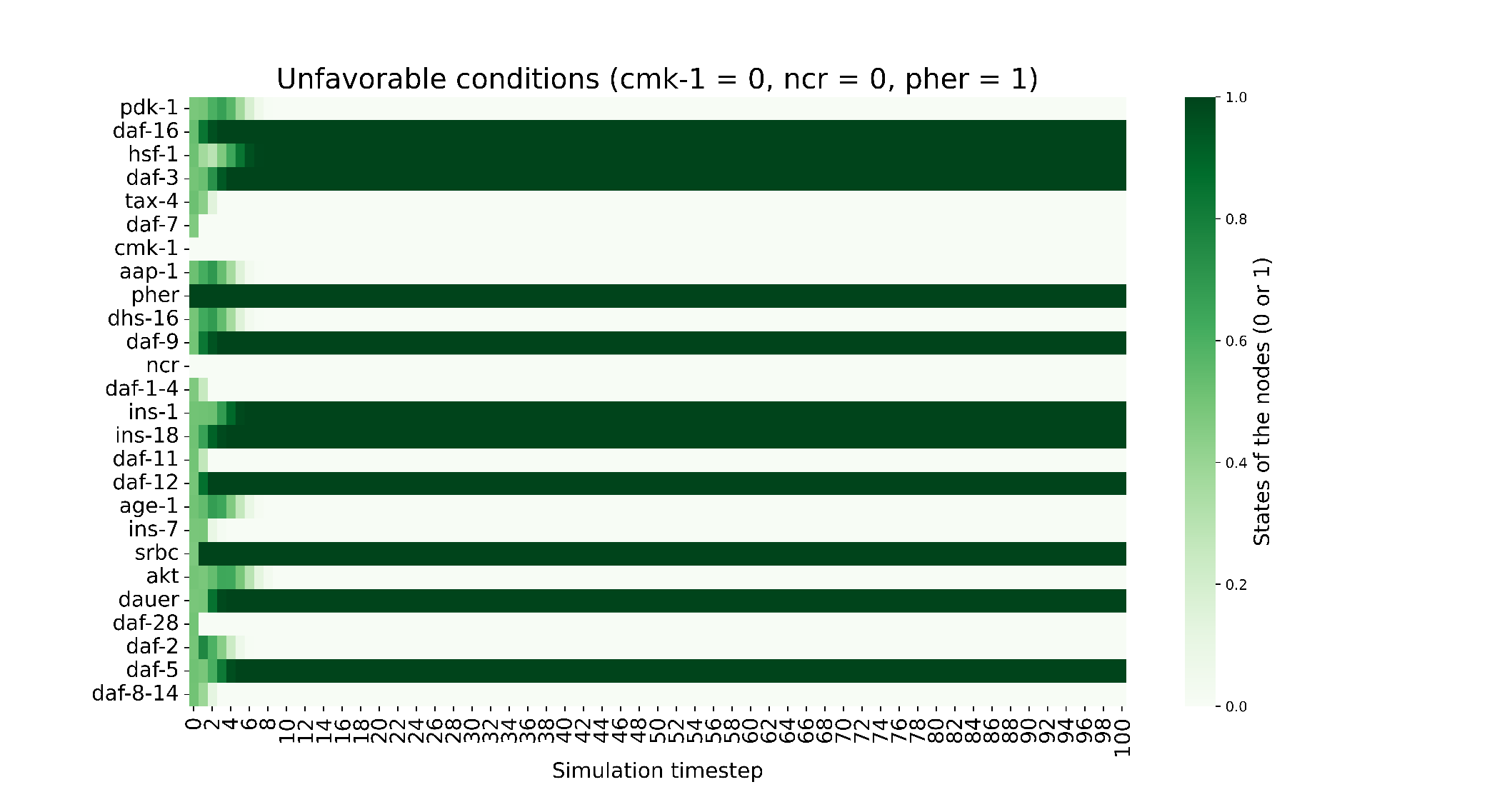


Simulation Trajectories for daf-d mutant models


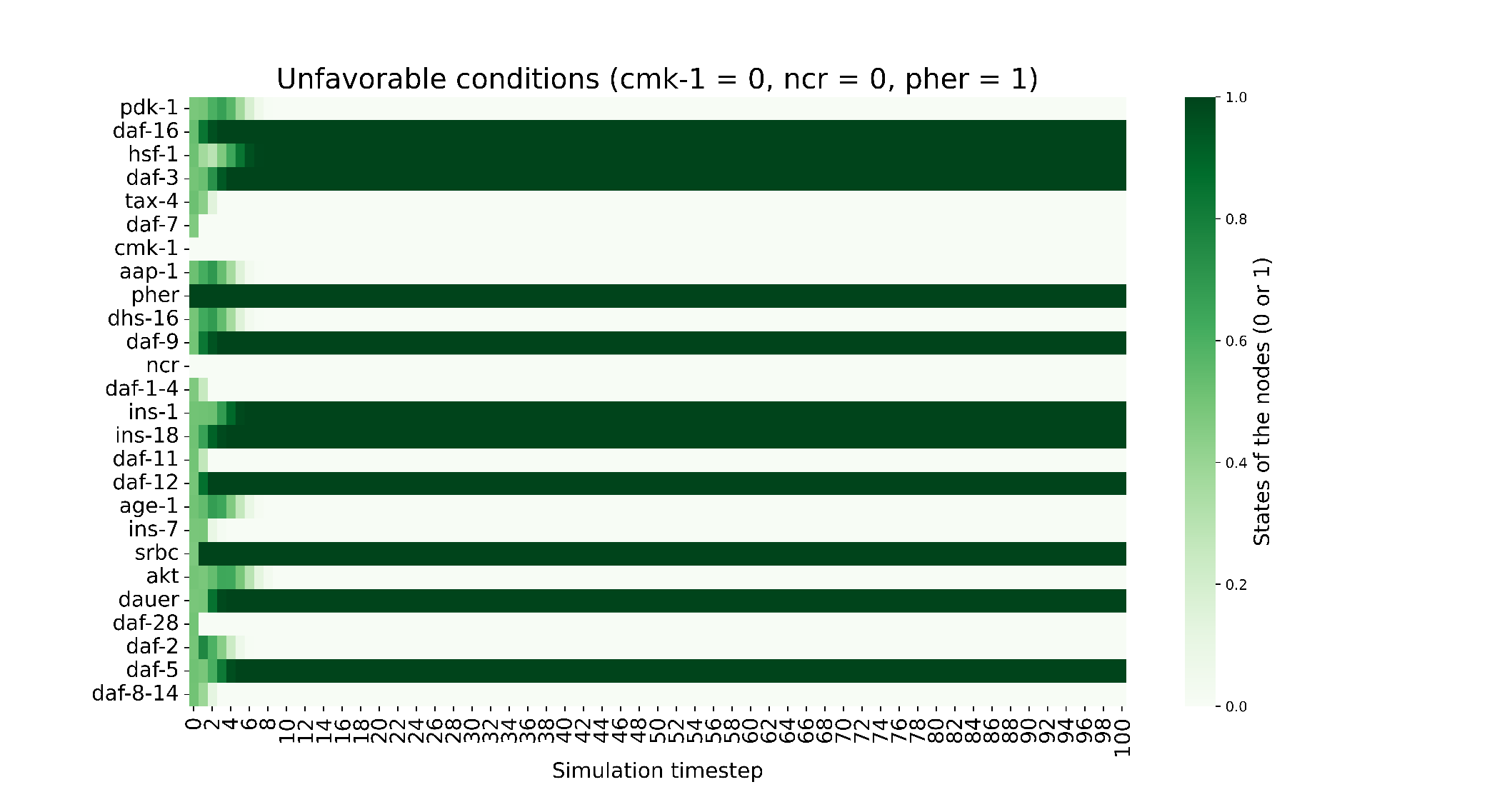

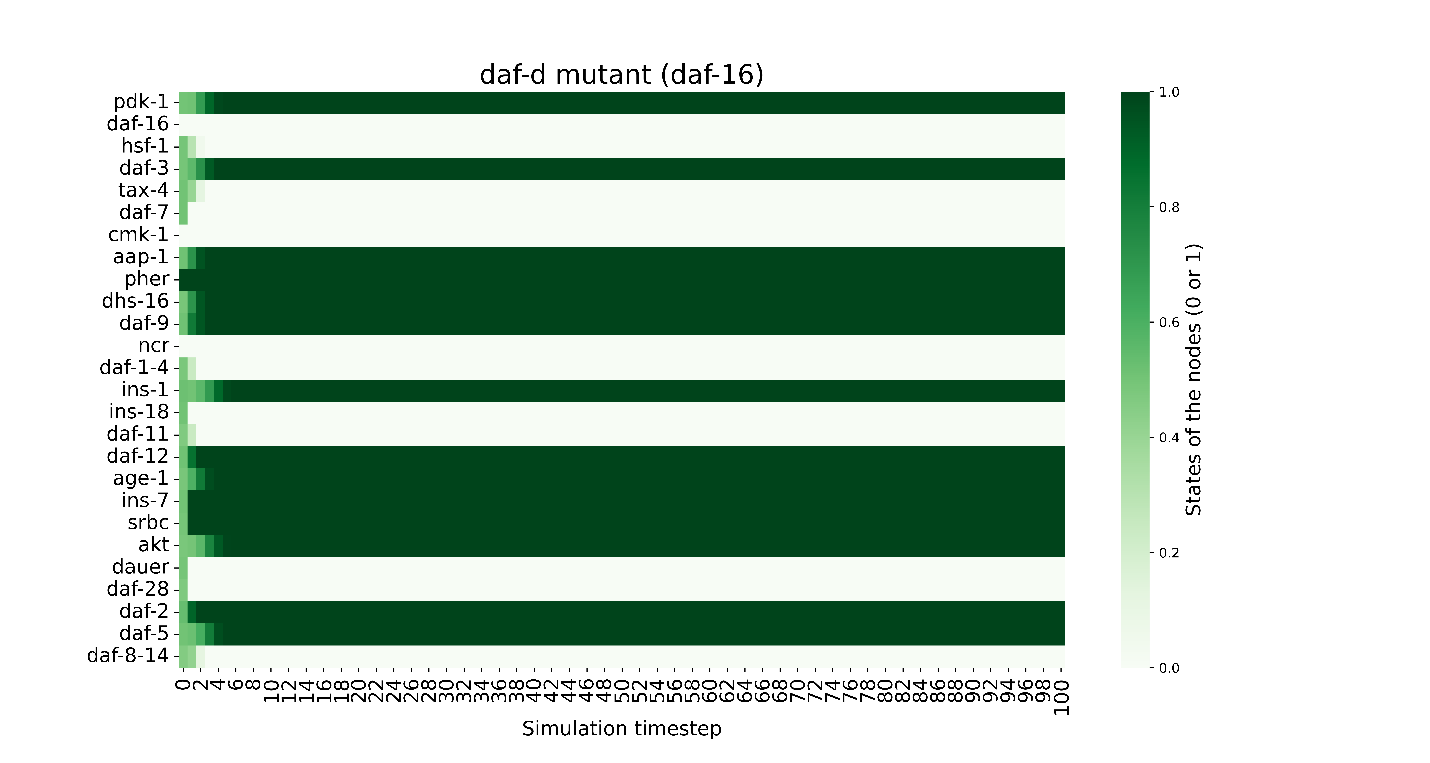

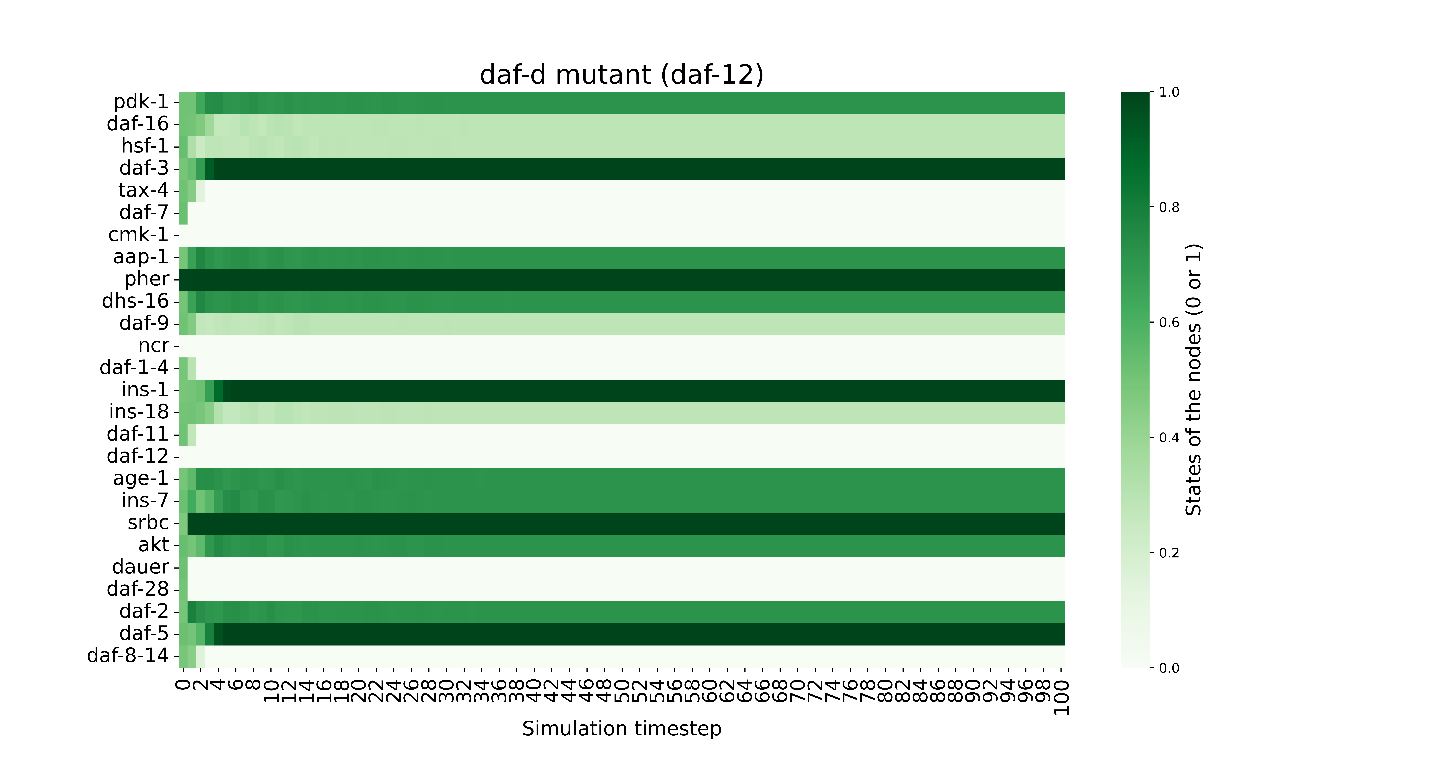

Supplement: Figure S2 — Simulation trajectories for wildtype, daf-d and daf-c mutants [file peerj-11-14713-s005.docx]
